# A Discrete Model of *Drosophila* Eggshell Patterning Reveals Cell-Autonomous and Juxtacrine Effects

**DOI:** 10.1371/journal.pcbi.1003527

**Published:** 2014-03-27

**Authors:** Adrien Fauré, Barbara M. I. Vreede, Élio Sucena, Claudine Chaouiya

**Affiliations:** 1 Instituto Gulbenkian de Ciência, Oeiras, Portugal; 2 Yamaguchi University, Faculty of Science, Yoshida, Yamaguchi City, Yamaguchi, Japan; 3 Universidade de Lisboa, Faculdade de Ciências, Departamento de Biologia Animal, Campo Grande, Lisboa, Portugal; Princeton University, United States of America

## Abstract

The *Drosophila* eggshell constitutes a remarkable system for the study of epithelial patterning, both experimentally and through computational modeling. Dorsal eggshell appendages arise from specific regions in the anterior follicular epithelium that covers the oocyte: two groups of cells expressing *broad* (roof cells) bordered by *rhomboid* expressing cells (floor cells). Despite the large number of genes known to participate in defining these domains and the important modeling efforts put into this developmental system, key patterning events still lack a proper mechanistic understanding and/or genetic basis, and the literature appears to conflict on some crucial points. We tackle these issues with an original, discrete framework that considers single-cell models that are integrated to construct epithelial models. We first build a phenomenological model that reproduces wild type follicular epithelial patterns, confirming EGF and BMP signaling input as sufficient to establish the major features of this patterning system within the anterior domain. Importantly, this simple model predicts an instructive juxtacrine signal linking the roof and floor domains. To explore this prediction, we define a mechanistic model that integrates the combined effects of cellular genetic networks, cell communication and network adjustment through developmental events. Moreover, we focus on the anterior competence region, and postulate that early BMP signaling participates with early EGF signaling in its specification. This model accurately simulates wild type pattern formation and is able to reproduce, with unprecedented level of precision and completeness, various published gain-of-function and loss-of-function experiments, including perturbations of the BMP pathway previously seen as conflicting results. The result is a coherent model built upon rules that may be generalized to other epithelia and developmental systems.

## Author Summary

We propose a modeling approach to *Drosophila* egg appendage development. Relying on a thorough compilation of published data, our model comprises cellular genetic networks and cell-cell communication. It proves to be extremely robust by faithfully replicating the wild type pattern as well as patterns arising from mutational perturbations. Our model proposes a new hypothesis for the definition of the anterior competence region, through early posterior EGF activity in conjunction with an early BMP signaling event, which may reconcile apparently conflicting experimental results. Furthermore, this is the first model that explicitly includes the removal of Grk, possibly through vitelline membrane formation, as a central event in patterning the follicular epithelium. Importantly, the model predicts the requirement of an instructive juxtacrine signal to specify the different regions of the developing appendages. Finally, our modeling framework may be applied to other developing epithelia and biological systems.

## Introduction

Models of morphogenesis have existed since, at least, the early 20th century when D'Arcy Thompson published his study on growth and form [1]. Mathematical abstractions to understand biological systems developed greatly during the next decades following the works of Turing and Meinhardt amongst others [2]–[4]. A primary stage for the test of much of these ideas has been *Drosophila melanogaster*, still today a favored model system in the interplay between modeling and experimental approaches to biology [5]–[13].The early embryo and the imaginal discs are classical examples of (ongoing) success stories in this dialog between the abstraction and heuristic properties of modeling, with the necessary testing and validation potential of experimental biology [14], [15]. Yet, many other developmental events may be amenable to this exercise. Here, we present a study on the development of eggshell structures in *Drosophila*, with emphasis on the dorsal egg appendages, which have been the object of important modeling efforts in recent years [9], [16]–[19].

**Figure 1:**
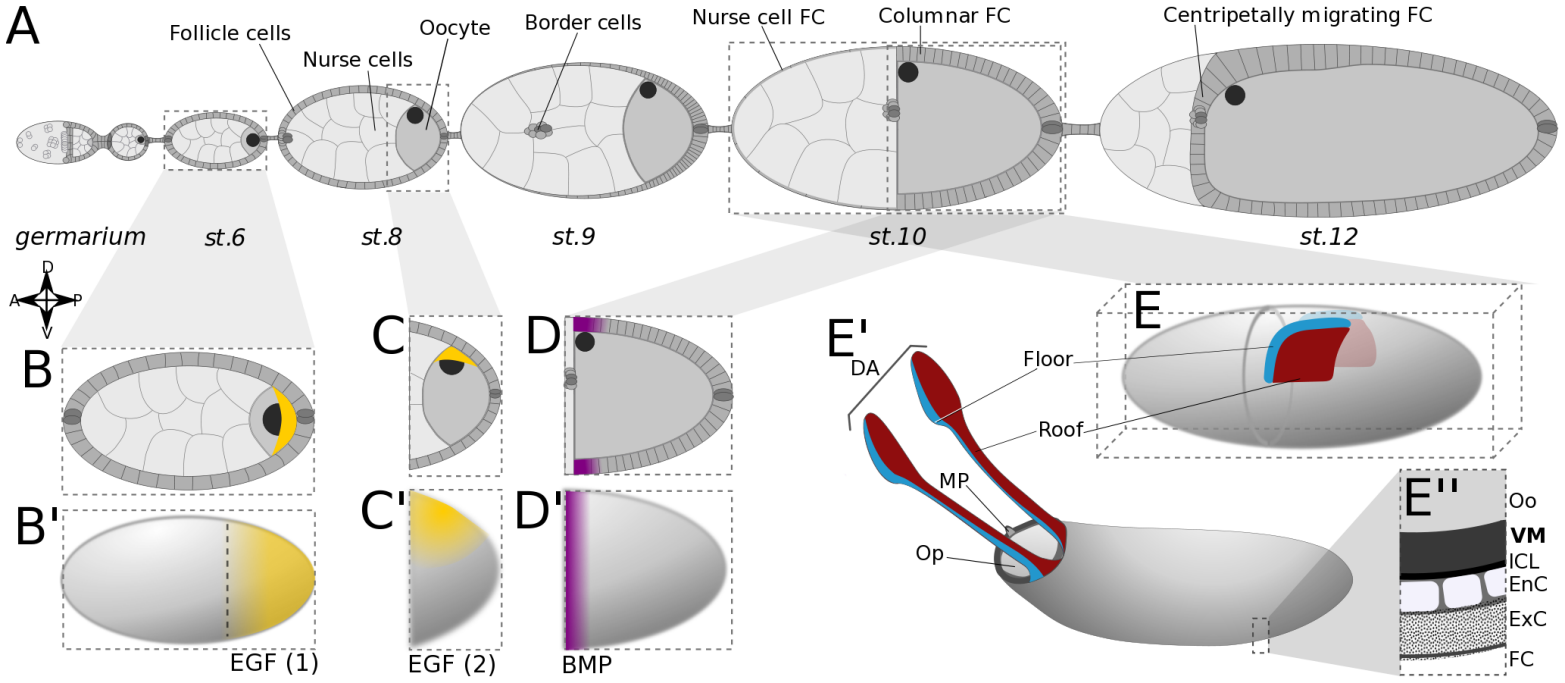
Overview of oogenesis in *Drosophila melanogaster*. (**A**) Schematic of an ovariole. Egg chambers, displayed at progressively later stages from anterior (left) to posterior (right), are formed in the germarium, and consist of three main cell types: nurse cells and the oocyte, both germ line, enveloped by a layer of somatic follicle cells (FC). After stage 9, the FCs have remodeled to form a columnar epithelium over the oocyte, and a squamous epithelium over the nurse cells. (**B–B′**) At early stages, ligand Gurken (Grk; in yellow) co-localizes with the oocyte nucleus to the posterior pole of the oocyte. It signals to EGFR in the overlying FC, activating the EGF pathway in a posterior-anterior gradient. (**C–C′**) After oocyte repolarization, Grk and the oocyte nucleus are located at the dorsal-anterior cortex of the oocyte. The EGF pathway is locally activated in overlying FC. (**D–D′**) Dpp ligand produced in the anterior FC establishes a steep anterior-posterior gradient of BMP signaling activity in the columnar FC. (**E–E″**) The appendage primordia are defined at stage 10 and consist, on either side of the midline, of two groups of cells, roof and floor. The eggshell deposited between the oocyte (Oo) and the follicle cells (FC) contains the operculum (OP), the micropyle (MP), and two dorsal appendages (DA); and is constituted by the vitelline membrane (VM), the inner chorionic layer (ICL), an endochorion (EnC) and an exochorion (ExC) [31].

The eggshells of *Drosophila* species are conspicuously oriented and patterned. Specialized chorionic structures include the dorsal-anterior operculum, through which the larva will hatch, and the dorsal appendages (DA), thought to facilitate the gas exchanges of burrowed eggs [20]. These structures arise from the follicular epithelium that surrounds the oocyte during the late stages of oogenesis (Figure 1A), based on a pattern established around stage 10. In particular, the two spots of *broad* (*br*) expression along the dorsal midline at stage 10B define the future appendage-forming regions [21]. More specifically, Br marks the roof of the DA, whereas the presumptive floor is associated with *rhomboid* (*rho*) expression (Figure 1E–E′) [22].

Two main signaling pathways are responsible for the anterodorsal patterning of the *Drosophila* eggshell: EGF and BMP [23]–[25]. In the EGF signaling pathway, the TGF-α-like ligand Gurken (Grk) originates from the oocyte, and signals to EGF receptor (EGFR) in overlying follicle cells. Early signaling takes place at the posterior pole until stage 7–8, when Grk moves with the oocyte nucleus to the dorsal-anterior corner (Figure 1B–B′) [26], [27]. Here, Grk activates EGFR in dorsal follicle cells until stage 10B, when the formation of the vitelline membrane (VM) creates a putatively impenetrable barrier (Figure 1E″) [16], [28]. The BMP gene *decapentaplegic* (*dpp*) is expressed from stage 8 onwards in an anterior subset of FC (Figure 1C) [29], and encodes a ligand for BMP type I and II receptors expressed in the follicular epithelium [30], [31]. Both pathways have been shown to co-regulate the expression of *br* and pattern the eggshell [23], [32], through a genetic network in which numerous genes have been implicated; either directly as mutant phenotypes, indirectly as EGFR targets, or because of their differential expression patterns in the dorsal domain [33], [34]. Moreover, studies have associated other signaling pathways such as JNK, Ecdysone, and Notch [35]–[40]. In the face of such overwhelming complexity, computational modeling constitutes an important and complementary approach to understand the cellular and molecular underpinnings of *Drosophila* eggshell patterning.

In the past 15 years several computational models have been proposed, building on an increasingly sophisticated insight of oogenesis. A first system was proposed when Wasserman and Freeman showed how a two-peak pattern of EGFR activation along a one-dimensional lateral axis could be formed through positive and negative feedback loops with different thresholds [16], a concept mathematically explored by Shvartsman et al. [41]. Subsequently, further research into eggshell pattern formation [42] led to a revised model with a distinct network-based approach, designed by Lembong et al. [9]. However, still only one dimension was considered in this model, relying solely on the dorsal-anterior Grk signal to set the posterior limit of the *br* expression domain. When the same network was applied to a two-dimensional surface, it appeared that a second signal was required to obtain correct pattern formation [18].

The identity of this second signal is controversial. Evidence supports the idea that BMP signaling is a requirement for the definition of an anterior DA competence region [23], [31], [43]. However, work by Yakoby et al. [34] contradicts this role of BMP signaling, and the most recent models [18] endorse the view that the posterior border of the competence region is set solely by early posterior Grk signaling. This hypothesis is strongly supported by recent experimental data [24]. This leaves unexplained the evidence for BMP pathway involvement in defining the anterior competence region, as well as the conflicting experimental results on this matter. Further modeling may help reconciling these observations under a unifying framework.

One open question is the specification of the single-cell wide, L-shaped floor domain, recently tackled by Simakov and colleagues [19]. Using a two-dimensional hexagonal grid, they postulate a juxtacrine signal emanating from the anterodorsal-most region of the epithelium. However, the underlying network departs in several ways from published genetic interactions. For example, the known cell-autonomous activation of Rho by the EGF pathway [44] is instead described as an inhibition (via a hypothetical factor G4) and, similarly, the cell-autonomous inhibition of Rho by Br [22] is defined as an activation (via G4). Moreover, important deviations in the resulting expression patterns can be observed relative to the experimental data. For instance, the final pattern of Pnt (called G1) that results from this model differs from the published data, in that it should abut the Br (G3) pattern [42], [45], [46]. Discrepancies also appear in the clonal simulations, in particular regarding the position of the Rho (G2) and Br (G3) cells with respect to the clone boundary (compare with Ward et al. [22] and Boisclair-Lachance et al. [45]). Both these differences point to an issue with the specification of the floor domain, for which we would like to propose an alternative hypothesis.

Thus, we here present a new model of *Drosophila* eggshell patterning, using a hierarchical, qualitative framework that combines experimentally supported intracellular networks and cell-cell interactions in an epithelial context. A thorough review of the existing data on eggshell patterning is at the basis of our work. In tune with Simakov and colleagues [19], we define the epithelium as a grid of hexagonal cells and postulate the action of a juxtacrine signal in pattern formation. However, we propose that this signal stems from the putative roof cells, and not from the operculum as suggested by Simakov and co-workers [19]. Furthermore, we hypothesize that this signal acts through amplification of the EGF signal in the floor domain.

With regard to the controversy surrounding the influence of BMP pathway activity on DA formation, we suggest that the conflicting data can be reconciled by postulating an early BMP pathway signal that acts synergistically with the EGF-controlled mechanism identified by Fregoso Lomas et al. [24]. These pathways may cooperate in early stages to define the anterior DA competence region.

Our first approach is a simple, phenomenological model that solely incorporates the roles of the two main signaling pathways (EGF and BMP) within an anterior competence region during late (stage 10) eggshell patterning, and a juxtacrine signal sent by the roof cells. Building on this concept, and using an experimentally substantiated genetic network, we then propose a detailed mechanistic model. Importantly, this is the first model to demonstrate the importance of Grk signaling extinction in achieving the final pattern. Furthermore, extensive simulations of mutants and clonal analyses provide a systematic test against published data.

Finally, we implemented a simple, discrete modeling framework that integrates logical models of cellular regulatory networks onto epithelial grids. This allows the consideration of both intra-cellular and extra-cellular signaling. This approach goes along the same line as other work defining epithelium models by integrating single-cell models [47], in particular through the use of Boolean models [48].

## Results

In this study we rely on a hierarchical framework that integrates single-cell models, defining qualitative intra-cellular regulatory networks, into epithelial models, where cells are interconnected within a grid (see Methods). Given the complexity of the molecular network, we first model the system from a phenomenological standpoint. Despite its simplicity, this model is able to recapitulate wild type dorsal follicular epithelium pattern formation with great accuracy. Building upon this result, we proceed to the assembly of a genetic network based on experimental data. The resulting mechanistic model proves to be effective both in wild type and various mutant scenarios.

### Phenomenological model

#### Defining logical rules in a single-cell context

We define a single-cell model with three essential output fates: roof, floor, and operculum, represented by Boolean variables. *In vivo*, the floor and roof regions combine into an appendage primordium on either side of the dorsal midline, while the presumptive operculum occupies most of the dorsal epithelium anterior to the dorsal appendages (DA) (Figure 1E–E′). These three domains form within an anterior competence region, the definition of which will be addressed in the next section. For now, we simply represent it by a Boolean “anterior” variable.

Within this anterior domain, EGF activity is required for the formation of all three types of tissue. The roof primordia require low levels of EGF activity, but are repressed by high levels of EGF and by BMP activity (Figure 1C,D) [23], [32], [34]. EGF is thus represented by a ternary variable; BMP activity, by contrast, is Boolean.

Operculum fate is assigned to cells receiving either high levels of EGF, or both EGF and BMP signaling. The logical rules controlling the activity of these variables stem largely from an interpretation of pattern formation in a two-dimensional epithelium. To define the floor cells, a single-cell wide domain is required. Similar to what was proposed by Simakov and colleagues [19], we postulate that the floor domain forms at the interface between roof and operculum. However, in contrast with this work, we propose a mechanism whereby floor cells are on the operculum side of this border, and set for floor formation a similar rule as for operculum fate, with the additional requirement that it is in contact with a roof cell. This rule arises from the observation that the boundary between roof and floor is sharp, while floor and operculum overlap [49].

In our model this hypothetical juxtacrine effect is achieved through the variable Roof_adj, which is set to 1 when a neighbor is a roof cell. Analysis of the stable states of this single-cell model shows that the different input combinations of EGF and BMP within the anterior domain indeed lead to four possible stable states (cell fates): roof, floor, operculum, or main-body follicle cells (data not shown).

#### The single-cell phenomenological model in epithelial context

**Figure 2:**
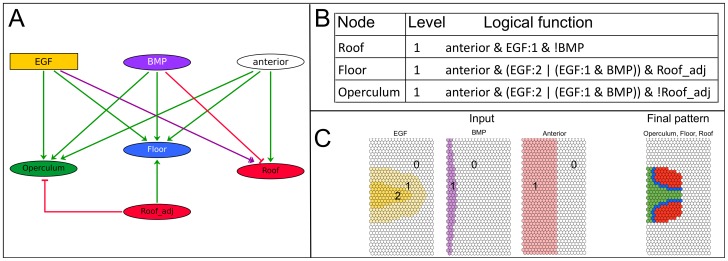
Phenomenological model: rules and result. (**A**) Regulatory graph: the model links three distinct follicle cell fates, Operculum, Floor and Roof, to a combination of input components EGF, BMP, *anterior*, and Roof_adj. Oval nodes are Boolean (0 or 1) and the rectangular node (EGF) is associated to a multi-valued variable, which here takes values between 0 and 2 (absent, intermediate and high level). EGF directly influences the position of the three domains on the dorsal-ventral axis. BMP establishes the anterior border of the roof, while *anterior* defines the anterior competence region. Roof_adj is an input variable accounting for the differentiated state of neighboring cells. Green and red edges denote positive and negative effects, respectively. The edge in purple denotes a dual effect, i.e. activating or repressing, depending on the level of its source. (**B**) Logical functions driving the dynamics of the model: Each rule specifies under which conditions the variable evolves to value 1 (otherwise, the variable tends to 0). The condition of the presence of EGF is simply denoted as “EGF”, and “EGF:1” or “EGF:2” whenever distinction between levels is required. Logical connectors are: & for a conjunction (and), | for a disjunction (or) and ! for a negation (not). (**C**) Epithelial model: left, patterns for the inputs EGF (yellow), BMP (purple) and *anterior* (pink) as used during the simulation. Right, final cell fates are shown in green (operculum), blue (floor) and red (roof).

Moving to a two-dimensional context, we define the corresponding *epithelial model*, where all cells contain the same set of variables. We consider static EGF and BMP inputs. At stage 10, Gurken (Grk) protein is observed as an elongated stripe along half the length of the dorsal midline [27],[50], while known targets of the EGF pathway are either activated or repressed in a broader dorsal domain [23]. The BMP gradient, by contrast, is steep: BMP pathway activity as detected through pMAD, is strong in a thin band of cells in the anterior region that is slightly wider at the dorsal side [42]. The anterior region is set so it matches the posterior limit of the roof domain [43], [35] (Figure 2C, left panel). In spite of the crudeness of the model, the simulation successfully replicates wild type pattern formation (Figure 2C).

This encouraging result supports the hypothesis of a juxtacrine signal originating from the roof region as a key mechanism in the specification of the floor, which we set to explore in the next section.

### Mechanistic model

**Figure 3:**
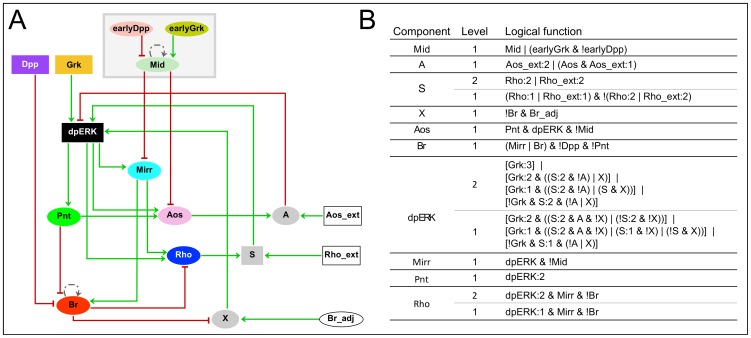
Mechanistic cellular model. (**A**) Regulatory graph: Grey nodes combine para- and/or autocrine signals. Dotted edges on Br and on Mid represent *ad hoc* interactions to account for protein maintenance. The grey box encompasses the module defining the anterior competence region (see text and Supplementary Text S1 for details). Other graphical conventions are as in Figure 2. (**B**) Logical functions driving model dynamics: (see Figure 2 for notation).

With the framework set by the previous phenomenological approach, we replace the different abstract cell fates and the “anterior” domain by relevant genetic markers as to provide a genetic basis for the network linking the model's input pathways to its output. Figure 3A shows this network, designed chiefly upon experimental evidence (see additional details in Supplementary Text S1).

#### Defining the anterior domain

As recently shown by Fregoso Lomas and colleagues, the posterior border of the anterior competence region is set by early EGF signaling at the posterior pole, mediated by expression of the transcription factor Midline (Mid) in the posterior region [24]. In addition, there is evidence that BMP signaling also plays a positive role in defining the anterior region: it has been shown that *decapentaplegic* (*dpp*) overexpression expands the anterior domain towards the posterior [32], [43], and disruption of BMP signaling in mutant clones suppresses the expression of the roof marker Broad (Br) in a cell-autonomous manner [23], [43]. Yet, these results have been contradicted by Yakoby and colleagues, who found that disruption of the BMP pathway triggered Br expression in the anterior most region, but had no effect in the presumptive roof domain [42].

A possible explanation for this difference can be found in the size of the clones: the mutated domains shown in the publication of Yakoby and colleagues [42], are much smaller than the mutant cell populations in both papers from the Roth lab [23], [43]. Furthermore, in a recent publication a similar analysis was performed with the BMP type I receptor Wishful thinking (Wit), where large sized clones mutant for *wit* also lack high levels of Br expression [31]. Since follicle cells stop dividing around stage 7 [28], [51], clone size indicates a difference in timing of the onset of mutation prior to this stage. While visible *dpp* expression only starts at stage 8, there are several indications that activity in the BMP pathway indeed occurs prior to stage 7, and thus could explain these contradicting results of clones induced at different times during oogenesis. For example, clones mutant for *Mad* show ectopic expression of *brinker* in early (stage 6) egg chambers [52].

Therefore, we postulate a thus far unidentified early BMP signal, either activated by Dpp or another BMP ligand, which is required for the establishment of the anterior competence region. This signal could work through repressing or otherwise restricting Mid. We modified our model to include an earlier stage of pattern formation, which establishes the anterior competence region. Two new input variables were introduced to represent early EGF and early BMP signaling, and we defined the “anterior” variable as the absence of the posteriorly located Mid (grey box in Figure 3A).

#### The genetic network

Downstream of Mid, Grk, and Dpp, key nodes of the network are Br and Rhomboid (Rho), markers of the roof and floor domains, respectively [22], [53]. The genes are known to interact: Br represses *rho* transcription [22], and Rho, a protease, indirectly activates EGFR by cleaving its diffusible ligand Spitz to an active, secreted form [54]. It has been shown that Rho is required to maintain the late EGF activity in the roof cells [17], [55]. Moreover, *rho* itself is a transcriptional target of EGF signaling, via the transcription factors CF2 (not explicitly considered in the model) and Mirror (Mirr) [44], [56]. Both Rho and Br have been shown to display distinct levels of expression [21], [34], [57].

As for Br, it is indirectly targeted by EGF activity via the transcription factors Mirr and Pointed (Pnt) [46]. Both genes are downstream of the EGF pathway; their expression patterns, in respectively wide and narrow dorsal domains, suggest that Mirr responds to low levels of EGF activity while Pnt requires high levels [56], [58]. *br* expression is controlled by Mirr and Pnt, through two distinct enhancers [46]. In our model we consider only the BrL enhancer that drives high-level *br* expression in the roof, which is activated by Mirr and repressed by Pnt, thus explaining the contrasting effect of EGF activity on roof specification along the dorsoventral axis [46]. As early low-level Br through the BrE enhancer is insufficient to inhibit *rho*, as evidenced by their co-expression at stage 9, we cannot find a role for BrE in our patterning network and have omitted low-level Br in our model.

EGF activity is represented in the model by dpERK, the phosphorylated form of ERK (MAPK), which is part of the EGF signal transduction cascade. Levels of dpERK are modulated by several factors, including Argos (Aos), a secreted protein that sequesters EGFR ligands Grk and Spitz [59]. dpERK in turn induces *aos* [16], through as of yet undetermined factors. Two known regulators of *aos*, active in other tissues, are also present in the follicular epithelium: Capicua [60], which responds to low levels of dpERK activity and also regulates *mirr* (not shown in the network for the sake of simplicity); and Pnt [61], which like *aos* itself responds to high levels of dpERK activity. In absence of better evidence for *aos* regulation, we have included the latter pathway as a working hypothesis.

The BMP pathway has been shown to set the anterior boundary of Br: around stage 10, Dpp signaling in the anterior-most rows of the columnar epithelium inhibits *br* expression [32], [42]. Finally, based on evidence that expression of both *mirr* [62] and *aos* [63] are restricted to the anterior competence domain, we set them both under the negative control of Mid.

#### The mechanistic model in a single-cell context

From the above, six components constitute the core network of our model: below Mid, which integrates the influence of early Grk/EGF and BMP, and (late) Grk and Dpp, we find dpERK, Mirr, Rho, Aos, Pnt, and Br as their targets. We use Boolean variables to represent gene expression, unless finer description is required to account for a particular mechanism. Thus, early BMP, early Grk/EGF, Mid, Dpp, Mirr, Pnt, and Aos are all Boolean. We also use a Boolean variable to represent only high-level Br (as driven by BrL). Rho is ternary, considering the possibility that even low levels may have an impact on EGFR activity.

Finally, we define two positive levels of dpERK to distinguish between the low and high levels required for *mirr* and *pnt* expression, respectively. Moreover, now that the EGF pathway is dynamically represented, we introduce an additional level for Grk, to make the gradient smoother.

The juxtacrine effect proposed in the phenomenological model suggests that some unknown component present in the roof region has an effect on neighboring cells, potentially driving floor cell fate. To model this effect we use Br, a *bona fide* marker of roof cells, to drive an unknown juxtacrine effector “X” in a neighboring cell. Thus, X is active when a cell that does not belong to the roof (Br = 0) is in contact with at least one roof cell (Br = 1).

Evidence suggests that EGF activity might be locally enhanced in the presumptive floor. Indeed, whereas Aos, considered a long-range morphogen [64], is required for shutting off the EGF signal in the presumptive operculum [17], it does not seem to affect EGF activity nor *rho* expression in the presumptive floor. We propose that this is due to a positive effect of the juxtacrine signal on EGF activity. Thus, in our model, dpERK can be inhibited by Aos, activated by Grk or Rho (via Spitz), and have its activity enhanced by X (when a non-roof cell is in contact with a roof cell).

#### Transition from single-cell model to epithelial context

To facilitate the transposition of the single-cell model to an epithelial context, we need to introduce variables representing the levels of Rho/Spitz, Aos, and Br in the neighboring cells. These will be Rho_ext, Aos_ext, and Br_adj, respectively. Rho_ext and Aos_ext integrate long-range information from the environment, while Br_adj receives input solely from direct neighboring cells. To combine these environmental signals with the information in the cell itself, we introduce the nodes S, A, and the earlier mentioned X, for Rho, Aos, and Br, respectively (Figure 3A). As discussed above, it appears that local Rho is sufficient to activate EGFR in the context of the floor region, even in the presence of Aos. Thus, as with Rho, we assign two positive levels to both S and extracellular Rho (Rho_ext), to reflect different levels of Spitz released by the cell itself or its immediate neighbors (see Supplementary Text S1 for details). In contrast, following the same reasoning, we consider that local Aos has a smaller effect, and needs the presence of paracrine Aos above a threshold to inhibit EGF activity. Thus, we assign one positive level for A and two for extracellular Aos (Aos_ext).

In a single-cell context, the logical rules can be inferred to a large extent from the wiring (Figure 3A) based on the experimental evidence presented above. This data, however, is not sufficient to set all parameter values, particularly for dpERK. In the context of this study and our juxtacrine mechanism hypothesis, we have set the remaining free parameters so as to maximize the effect of X. Finally, we introduce *ad hoc* self-loops on Br and Mid to make the patterns stable and facilitate analysis. These positive loops should not be interpreted as autoregulation, but rather as a way to translate into the model the sustained presence of protein in the cell.

The final set of logical rules is shown in Figure 3B.

### Mechanistic epithelial model

By assembling the single-cell models on a hexagonal grid, we obtain our final mechanistic epithelial model. In this epithelial context, we further need to include the spatial distribution of several factors. Both Rho (via Spitz) and Aos act at a distance, where Rho has a short range, and Aos a much longer-range effect. We thus define the variables Rho_ext and Aos_ext with equations containing the number of immediate or more distant neighbors expressing the corresponding variables (for details, see model documentation in the Supplementary Text S1). By contrast, variable X is easily defined, assuming that one Br positive cell is sufficient to trigger this signal in its non-roof neighbors. All other rules representing intracellular mechanisms can be transposed directly.

The variables are updated synchronously, with two specific exceptions. First, integration variables S, A, and X are systematically updated before all other variables. This is followed by dpERK, as variations in activity of the EGF pathway occur much faster than changes in gene expression, which is the case with the other variables. Second, we introduce a delay in Aos expression to account for the observation that its expression pattern does not immediately follow the changes in EGF activity.

**Figure 4:**
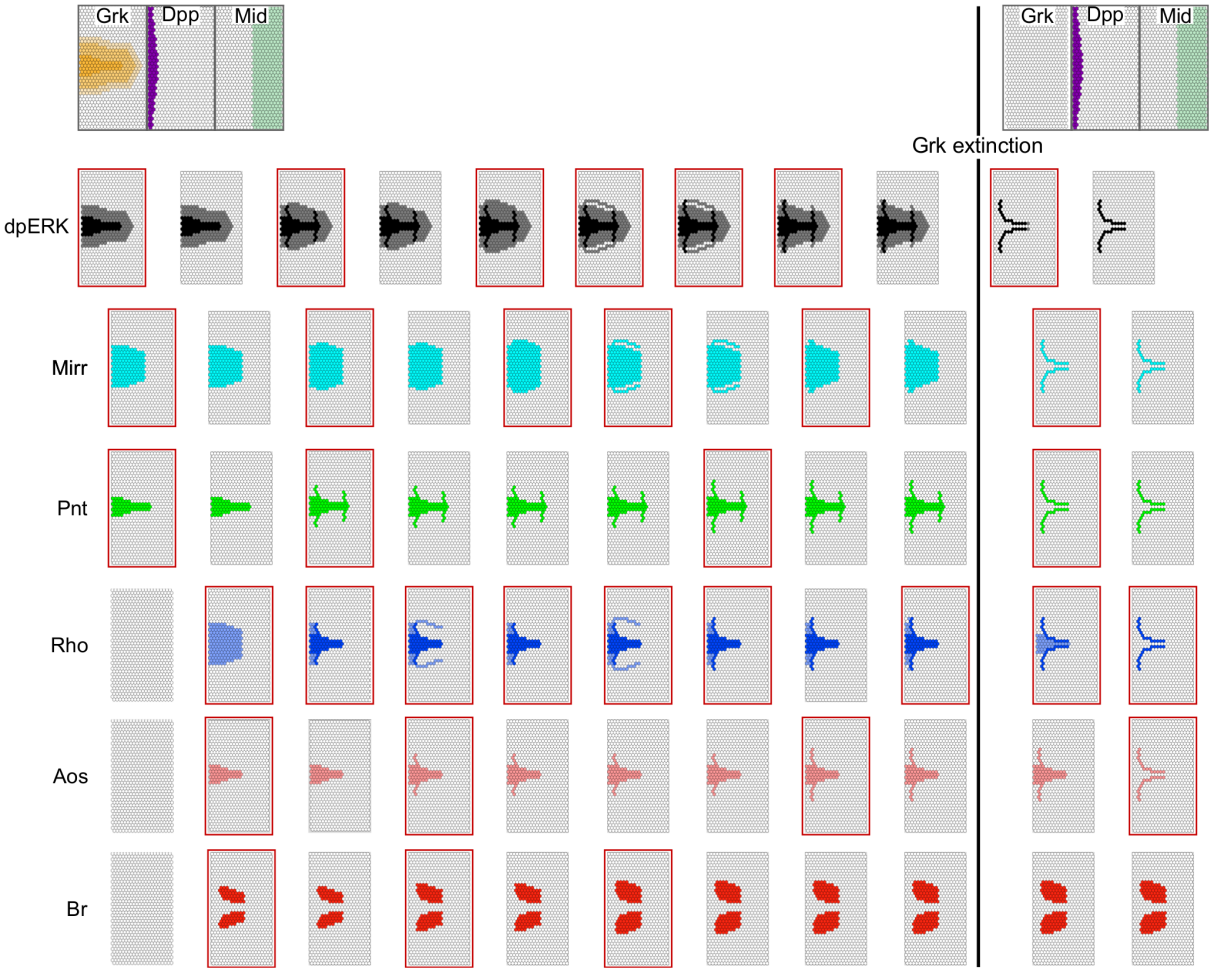
Mechanistic epithelial model, simulation. The simulation starts with a naive configuration (i.e. all cells are undifferentiated) upon which Grk, Dpp, and Mid input levels are applied. All components are updated synchronously except dpERK and the integration variables (not shown), which are always updated earlier (see text). From left to right are depicted the successive states of each component in the epithelium (gene expression patterns), before Grk extinction. The right panels show the components' states after Grk extinction. Color intensities are used for multi-valued components (Grk, Rho and EGRF, see Figure 3B). Red frames denote pattern changes.

With that in hand, we verified that the model performs consistently with the phenomenological model (Figure 2C) in reproducing the wild type patterns. Figure 4 depicts the step-by-step simulation, for the combination of inputs shown in the top left corner. The successive states are in agreement with experimental data, including the “spectacles” pattern of *rho* expression and EGF activity noted by several authors [17], [65]. When the simulation reaches a stable state, Br pattern matches the roof pattern obtained in the phenomenological case. However the patterns of Rho, Pnt and Aos conspicuously cover both the presumptive operculum and floor domains (compare with Figure 2C). The Mirr domain, by contrast, covers a larger region overlapping with all three presumptive domains.

To solve the discrepancy between this result and the experimental data, we introduce an as of yet disregarded player: the withdrawal of Grk, putatively mediated by the vitelline membrane (VM). The VM forms during stage 10, as the most important events in dorsal patterning unfold [28]. It has been hypothesized that VM formation effectively separates the oocyte, including the Grk signal, from the overlying epithelium [16]. It should be noted that other mechanisms, such as the degradation of the Grk signal over time, might also partly be responsible for the same effect. In any case, to the best of our knowledge the consequences of Grk withdrawal on gene expression in the epithelium have not been considered so far.

To include this event in our model we now set the Grk signal to 0 in the whole epithelium, and resume the simulation. The final patterns (Figure 4, rightmost column) recapitulate experimentally established wild-type expression patterns corresponding to epithelial domains giving rise to roof and floor of the DA. We note, however, that *in vivo aos* expression does not reach the floor pattern before stage 11 [66] to stage 13 [16], [65]. Thus, the delay associated to Aos is not sufficient to fully recapitulate the relative stability of the Aos pattern. Nevertheless, these results are consistent with the hypothesis that Grk signal disappearance is a crucial event in eggshell patterning.

### Assessing the robustness of the mechanistic model

In the epithelial model, the synchronous update of almost all variables reflects similar delays associated to the underlying mechanisms [67], [68]. To validate this choice, we performed a complete reachability analysis of the single-cell model under an asynchronous update, checking which stable states (cell fates) could be reached from an initial state, while generating all potential trajectories (see Methods section). This *in silico* experiment consists of inserting a cell at specific locations of the epithelium (thus specifying the values of input components of the single-cell model) and observing the resulting fate.

**Figure 5:**
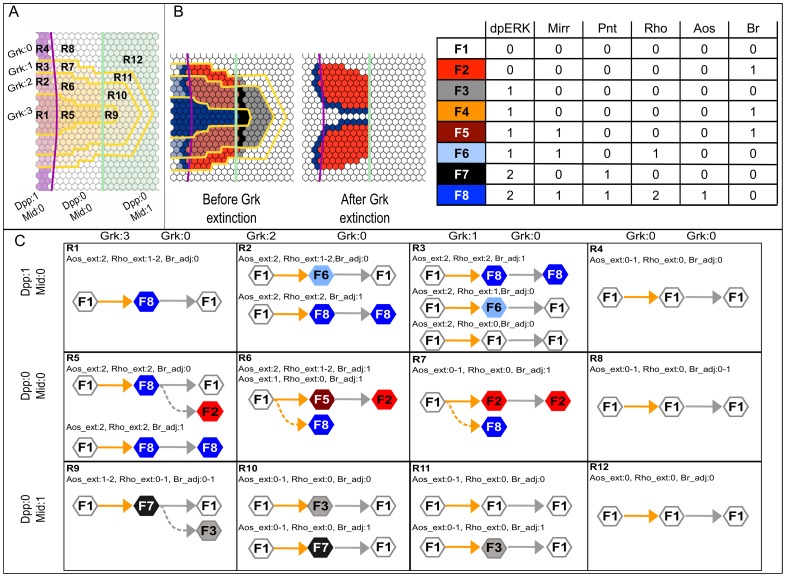
Mechanistic model tested. (**A**) Schematic dorsal view of the follicular epithelium, showing the 12 regions defined by combinations of input levels: Grk (4 levels, 0 to 3), Dpp (2 levels, 0 and 1), and Mid (2 levels, 0 and 1). (**B**) Final patterns before and after Grk extinction and description of the stable fates (F1 to F8). In regions R1 to R12, cells may adopt one of eight fates (F1 to F8) according to the values of the genetic network components (dpERK, Mirr, Pnt, Rho, Aos and Br; see also Supplementary Figure S1). The left diagram shows the final patterns obtained before and after Grk extinction (see Figure 4). Each row of the table describes the expression state of each component for a given fate. (**C**) Reachability analysis under the asynchronous update. In each region, we simulate the behavior of a naive cell inserted into the epithelium in its configuration just before the Grk extinction (the position of insertion determines the input values) and determine which stable state is reached (yellow arrows). The fate adopted by the cell follows the color code indicated in panel B. Upon Grk extinction, the simulation starts from a cell carrying the cell fate of the previous phase with now Grk levels set to 0, possibly leading to a new cell fate (grey arrows). In a few cases, more than one solution is attainable, such as in R5, R6 and R7. Full arrows represent trajectories towards fates matching the wild type situation, and dotted arrows indicate trajectories leading to alternative fates: e.g in R6 and R7, in addition to F5, the Br expressing pattern F8 is also reachable, unless a delay is assigned to Pnt (see text).

The different combinations of Dpp, Grk, and Mid levels partition the follicular epithelium into 12 regions, R1 to R12 (Figure 5A); the values of the remaining inputs further subdivide these regions. However, the 288 input combinations (levels of Dpp, Grk, Mid, Aos_ext, Br_adj and Rho_ext) yield only eight stable cell fates, named F1 to F8, and three oscillatory attractors (see Supplementary Figure S1). F1 corresponds to an undifferentiated state, either that of all follicle cells prior to the patterning process, or that of main-body follicle cells at the end of the process; F2 is reminiscent of the roof expression pattern; F8 corresponds to both the presumptive operculum and floor regions in early stages, or in the floor regions alone after the split. The other fates are similar to these three. For instance, F6 is similar to F8 in that it is positive for Mirr and Rho, but here *br* repression is due to high levels of Dpp instead of Pnt; so F6 only occurs in regions where Grk and Rho_ext are too low to induce high levels of dpERK and thus of Pnt.

We simulate the behavior of a naive cell, for fixed input values defined by the latest state of the epithelial model before and after Grk extinction (Figure 5B), in relevant (sub)-regions of the grid (Figure 5C).

We observe that in 10 out of the 12 regions, before Grk extinction, a single cell fate is reachable, consistent with the wild type simulation (Figure 4). Some regions (R2, R3, R10 and R11) are sub-divided regarding neighboring regions that include Br expressing cells; the reached fates match experimental observations. Also, in R6 and R7, where the roof should be formed, two attractors are reachable, but, reassuringly, they include fates F2 and F5, corresponding to the expected Br positive fates (Figure 5C). Interestingly, in these regions, the alternative fate F8 is not reachable if we introduce a delay in Pnt activity. Biologically, this delay may correspond to the respective phosphorylation and expression of two Pnt isoforms that comprise Pnt activity [58], [69].

We proceed with the reachability analysis subsequent to Grk extinction, starting from the cell fate reached by the epithelial model (Figure 5B). Under this test, the single-cell model, and thus our core network, performs exceedingly well (Figure 5C). Virtually all (sub)-regions, with the exception of R5 and R9, resolve into a single fate that matches the expression patterns observed in egg chambers at this stage of development, thus revealing high robustness. More importantly, a new sub-region emerges within R5. This is a single-cell domain, Rho positive, framing the Br positive domain along the midline and to anterior. This corresponds faithfully to the Br_adj component included to account for the *rho* expression pattern. It demonstrates that our modeling approach to that particularly difficult element of follicular epithelium patterning is efficient and robust at reproducing the biology of the system. We can verify that in the sub-region of R5 where Br_adj = 0, the appearance of the fate F2 (Br expressing cells) results from a competition between the degradation of Mirr (active in F8) and the synthesis of Br. Assuming that this synthesis takes more time prevents the reachability of the F2 fate. Finally, in region R9, although the system can reach F3 from F7 after Grk extinction, this cellular state will ultimately convert into the F1 fate, because dpERK cannot be maintained in the absence of sufficient Aos and Rho signals.

Thus, considering all possible relationships between delays, the long-term behavior of the simulation (the reachable attractors) is rather similar to that obtained in our epithelial model. Notably, even where a few alternative fates are reachable, the model never fails to include the correct cell fate. This confirms that the model is robust to variation in delays. Thus, the assumptions made concerning the delays do not affect the biological description and predictions generated by the model.

### The mechanistic epithelial model under mutational challenge

We test our model under mutational challenge to further assess the biological pertinence of its assumptions and, importantly, extend the scope of its predictions. We first simulate a series of experiments connected with the definition of the anterior competence region, so as to test the hypothesis that early BMP signaling plays a part in this process. We then proceed with a systematic assessment of the model's behavior under complete gain-of-function or loss-of-function mutants, followed by clonal analyses for the six core components of our model (Aos, Br, EGFR/dpERK, Mirr, Pnt, and Rho), plus our hypothetical X. These genetic analyses have been used extensively in the past decades, providing a wealth of experimental data to be compared to the outcomes of our model. While these comparisons are mostly favorable, it is important to note that it is difficult to precisely derive gene expression patterns from morphological descriptions, and vice versa, and that these results should be confirmed by further experiments.

#### Perturbation of the anterior competence region

**Figure 6:**
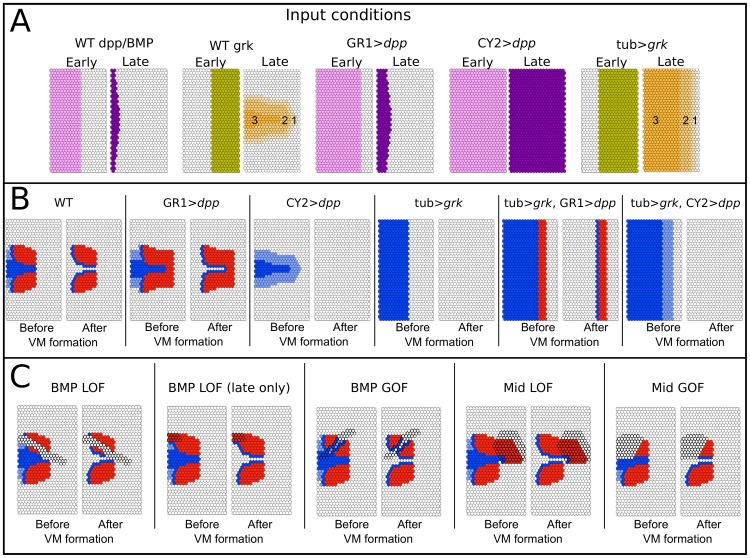
Perturbations of the anterior domain. (**A**) Simulation of wild type inputs, and of *dpp* mild and strong overexpression (using GAL4 drivers GR1 and CY2) [43], [63], [75], [83], and *grk* overexpression (using the Tub Gal4 driver). Asymmetry is maintained in GR1 and Tub driven inputs, taking into account the cumulative effect of the Gal4 driver and wild type expression. (**B**) Simulation results, pre- and post-Grk extinction, obtained from combinations of the input conditions described above. The boxes show the resulting patterns of Br (roof, red) and Rho (floor, blue). Compare to Shravage et al. 2007, Figure 3, panels Ea/Ec; Fa/Fc; Ga/Gc; Ha/Hc; Ia/Ic; Ja/Jc respectively [43]. (**C**) Perturbations of the BMP pathway and of Mid; LOF = loss-of-function; GOF = gain-of-function. BMP LOF was simulated by setting both the early_BMP and Dpp inputs to 0, to simulate a disruption of the BMP pathway before it could repress Mid; BMP LOF (late only) was simulated by setting only the Dpp input to 0, while keeping the early_BMP unchanged, to simulate a disruption of the BMP pathway after it could repress Mid. BMP GOF was simulated by setting both early_BMP and Dpp to 1 in the highlighted region. Mid LOF and GOF were simulated by setting Mid to 0 or 1, respectively, in the highlighted cells.

As discussed above, our model incorporates the notion that BMP signaling plays a positive role in the specification of the anterior region. Furthermore, we introduce the hypothesis that BMP activity acts at early stages of oogenesis by repressing Mid. Figure 6 shows the simulations we performed to test this hypothesis. To facilitate comparison with experimental results, the labels indicate the driver and genes of similar overexpression experiments [43]. First, panel A shows various inputs used for Grk and BMP (wild type as well as mild and strong overexpression simulation). Results of the simulations are shown in panel B, obtained using various combinations of input patterns; these can be directly compared to experimental work by Shravage et al. [43]. Most notable is how under mild BMP overexpression the Br spots expand towards the posterior and join over the midline region in a horseshoe-like pattern (Figure 6B). In a Grk overexpression background, mild overexpression of BMP produces a small central band of Br (Figure 6B). The complete absence of *br* in *grk* overexpression is also seen in experimental work [43]. Interestingly, while our model does not explicitly include operculum fates, in all simulations the Rho patterns obtained before Grk extinction fit well with experimental data on Fas3 patterns and operculum fate [43]. However, our model fails to reproduce the thin band of *br* expression observed with experimental CY2>*dpp* overexpression in both wild type and tub>*grk* background [43].

Panel C of Figure 6 deals with clonal perturbations of the BMP pathway and Mid. Importantly, we are able to simulate the effect of early versus late disruption of the BMP pathway. Simulations show that the former reproduces the results of Peri and Roth [23], Shravage et al. [43], and Marmion et al. [31], while the latter matches those of Yakoby and colleagues [42], thus reconciling apparently conflicting results. Our model predicts that constitutive activation of the BMP pathway in a clone would inhibit Br within the clone borders, while Rho appears around the new Br border. Whilst Midline overexpression in the anterior leads to the repression of the anterior fates in that domain, Mid loss-of-function in the posterior region leads to an expansion [24].

#### Whole-epithelium clones

**Figure 7:**
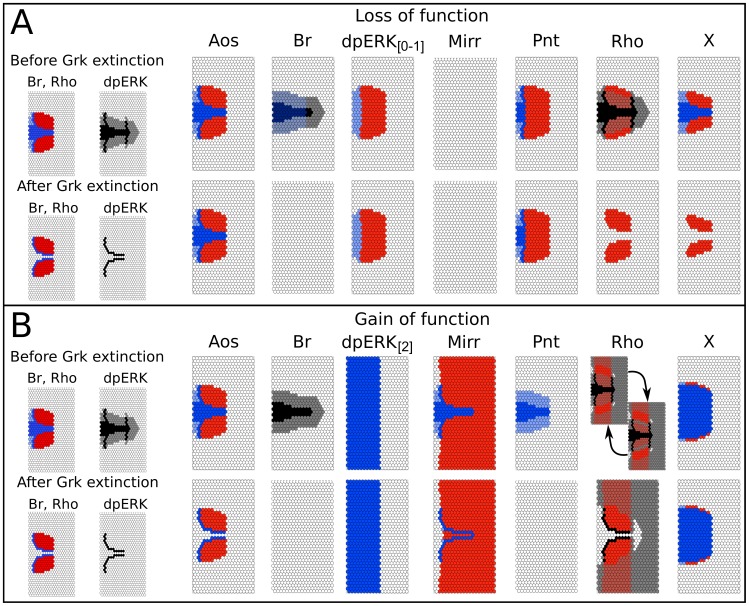
Mechanistic epithelium model. (**A**) Loss-of-function and (**B**) Gain-of-function analyses. Wild type patterns are shown on the left for comparison. Each of the boxes shows the resulting patterns of Br and Rho or dpERK, under gain-of-function or loss-of-function situations for multiple elements (corresponding to genes) of the model. The outcomes are shown both before and after Grk extinction on the top and lower rows of each panel, respectively. In the case of Rho GOF (for which the level is constrained between levels 1 and 2), a cyclic attractor is reached before Grk extinction, which resolves into a stable state afterwards (see text).

The simulations of loss- and gain-of-function mutants for the six core components of our model (Aos, Br, EGFR, Mirr, Pnt and Rho), as well as the hypothetical X, are presented in Figure 7.

Aos loss-of-function has no visible effect on the final Br patterns, but prevents the splitting of the dpERK pattern after Grk extinction, confirming experimental results [17], [45]. Our model predicts that this effect on dpERK is matched by a similar effect on downstream components, such as Rho. By contrast, simulation of *aos* overexpression does not have any effect on either Rho or Br domains, and thus fails to recapitulate the experimental evidence [70]. This problem could likely be solved by the introduction of a second, higher level of Aos, superior to the endogenous level and capable of stronger EGFR inhibition.

In the case of Br loss-of-function, our model initially shows an enlarged domain for Rho, Pnt, and Aos, associated with high dpERK. However, this pattern is entirely lost after Grk extinction. These results are consistent with the reported absence of DAs and an enlarged operculum in the Br mutant [21], [32]. Concerning *br* ectopic expression, our model predicts loss of Rho and limited EGFR activation, which is consistent with the dorsal appendage defect reported [21].

Given its central position in our network, it is evident that a complete loss-of-function of EGFR/dpERK would result in loss of expression of all the downstream markers. Instead, we have simulated partial loss-of-function by lowering the maximum level of dpERK activity to 1. This results in a phenotype very similar to loss of Pnt, and consistent with the reduction of the midline region: a fusion of the Br domains along with fused appendages has been reported in the literature (see [49] in particular). Constitutive activation of EGFR in our model (simulated by setting dpERK levels to 2 in all cells) results in the disappearance of the Br domain, and expansion of Rho, Aos, Mirr, and Pnt all around the anterior circumference, in almost perfect agreement with the experimental results [16], [42], [63]. Our model only fails to reproduce the patchy *aos* expression observed in the posterior domain by Queenan et al. [63]. This suggests that regulation may be subtler than what we have considered.

Loss-of-function of *mirr* results in a ventralized eggshell; conversely, ectopic *mirr* expression results in dorsalization [45], [56], [71]. Our simulations are consistent with these data, except for the reappearance of Br after Grk extinction in the midline. As Pnt disappears in the midline following the removal of Grk, the conditions for Br inhibition are removed as well. Meanwhile, overexpression of Mirr continues, inducing new Br-positive cells in the midline. It is possible that maintenance of the Pnt protein is relevant here, and would block Br reappearance. Regarding Pnt loss-of-function, our model correctly predicts the switch of the midline region to appendage- producing fate [45], [58], as well as the reduction of *br* expression in the Br domain following *pnt* ectopic expression [32]. Expectedly, Pnt gain-of-function entirely shuts off Br expression in the epithelium.

Finally, consistent with experimental data [45], Rho loss-of-function has no effect on the Br domains or on the width of the midline. However, after Grk extinction, loss of Rho results in the loss of EGF pathway activity and downstream gene expression in the presumptive floor, something that has yet to be shown experimentally. Simulation of *rho* ectopic expression yields a cyclic attractor in which cells oscillate between Br-positive and dpERK-positive states. These oscillations, generated by the dpERK-Pnt-Br-X-dpERK positive circuit can be considered artefactual as they are due to the synchronous activation and inactivation of Br and Pnt, something that is extremely unlikely to occur *in vivo*. These oscillations resolve in a single stable state, showing a ventral expansion of the Br domain that seems consistent with published observations [49], [72].

Overall, the results of our simulated perturbation experiments are in good agreement with the data reported in the literature, demonstrating the ability of our model to reproduce known effects of mutations. Thus, our model possesses a high predictive potential, which is of particular pertinence for the hypothetical factor X. As expected, absence of X causes a loss of the Rho domain after Grk extinction. Before Grk extinction, the effect of X mutation on the Rho domain is a lateral expansion of its anterior border. Interestingly, we observe a reduction of the Br domain, both before and after Grk extinction, along with the loss of the transient “spectacles” pattern of Rho expression and EGF pathway activity (not shown; see Figure 4 for the wild type case). By contrast, X gain-of-function results in a strong enlargement of the midline region, together with a reduction of the *br*-expressing region, implying smaller dorsal appendages that are further apart.

#### Partial clones

**Figure 8:**
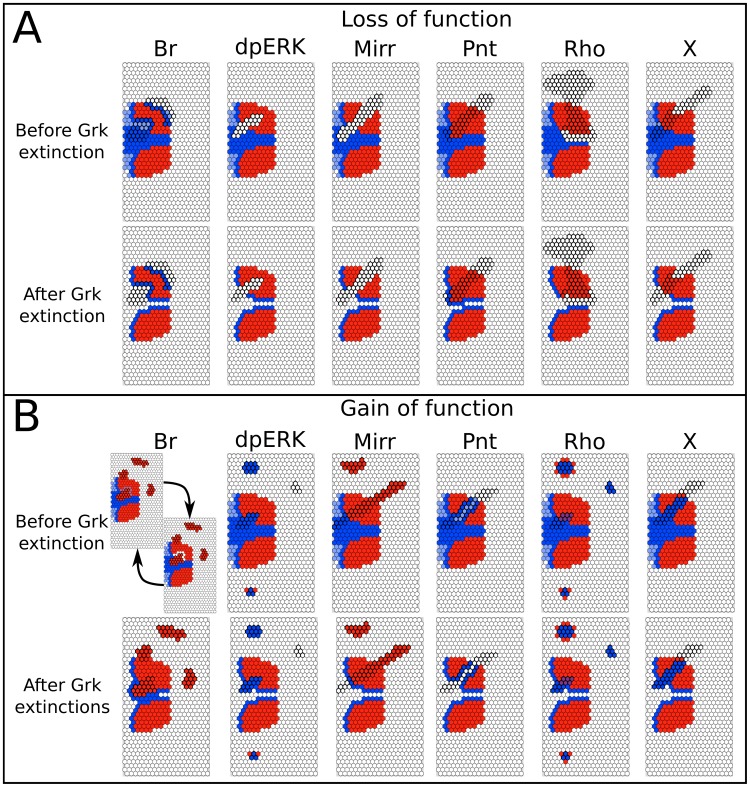
Mechanistic epithelium model, clonal analyses. (**A**) Loss-of-function clones. (**B**) Gain-of-function clones. Before Grk extinction (top row) and after Grk extinction (lower row). Row organization and color codes as in Figure 7. In the Br GOF case, the oscillatory attractor obtained before Grk extinction is due to the synchronous simulation scheme (see text). Here, we show the most consistent pattern of the two stable states resulting from the Grk removal.

Finally, mutant clone analysis puts the emphasis on cell-autonomous mechanisms (Figure 8). Br loss-of-function clones induce ectopic Rho-positive cells within the clone itself, a result consistent with experimental data [22]. This result illustrates the difference between our model and that of Simakov et al. [19], where similar clones (G3) lead to ectopic Rho (G2) expression outside of the clone's boundaries. Surprisingly, one of the Br gain-of-function clones in our simulation generates oscillations in the neighboring cells, which resolve into two stable states post Grk extinction. These oscillations, however, proceed from the same mechanism as previously discussed in the Rho gain-of-function case, and can be considered artefactual.

Disruption of the EGF pathway leads to loss of Br in the roof, consistent with the work of Yakoby and colleagues [42]. While we do not observe ectopic Br in the dpERK loss-of-function clones located in the midline, we expect that this expression may occur through the BrE enhancer, and its absence in our simulation reflects the modeling choice to include only high-level Br through the BrL enhancer [46]. Meanwhile, ectopic activation of the EGF pathway in the presumptive roof leads to cell-autonomous repression of Br. Additionally, in the ventral region, small, isolated anterior clones can also trigger non-autonomous Br in the neighboring cell, if distant enough from a source of Aos.

In the Mirr loss-of-function clones overlapping the roof region, we observe loss of Br, consistent with the reduced expression reported by Boisclair Lachance et al. in both the roof and midline regions [45]. Mirr ectopic expression in the ventral and posterior regions leads to ectopic Br in our simulations. Pnt loss-of-function in the midline results in an expansion of the Br domain [45], together with a corresponding displacement of the Rho border. Meanwhile, the observed cell-autonomous repression of Br in the Pnt gain-of-function clones mimics the ectopic activation the BMP pathway reported above. Late EGF activity in the floor is abolished in Rho mutant clones, whereas ectopic Rho in the ventral/lateral domain induces Br non cell-autonomously, but not Rho [45], [49]. Thus, in this case, we can only partially recapitulate experimental results.

Finally, our model predicts that localized loss of X results in a reduction of the Br domain in the lateral regions, together with a more significant loss of Rho within the clone's boundary at late stages. Ectopic activation of X results in cell-autonomous induction of Rho and disappearance of Br (through Pnt) in the dorsal-anteriormost region, and shows no effect outside of this domain.

## Discussion

In the face of the complexity of the regulatory network controlling dorsal eggshell patterning, it is difficult to isolate key players. While a handful of genes have been the focus of most experimental studies, dozens more have been implicated in eggshell patterning [34]. Moreover, a thorough examination of the literature reveals several inconsistencies: different authors report different patterns for the same gene, or similar patterns with different timing. The accuracy of our model in reproducing experimental data is therefore striking, in spite of its arguably simplistic view of the underlying molecular mechanisms.

While the model's predictions should function as a guide for experimentalists in further unraveling the mechanisms behind eggshell patterning, it also exposes inconsistencies in the current literature that deserve attention. For example, reports on *rho* expression vary significantly: at stage 10A, *rho* pattern is described as covering the dorsal area [26], [73], as displaying a spectacles shape [65], or as the two-striped domain of the roof [72], consensually established for later stages. Others still, have reported the beginning of *rho* expression to stage 10B [74].

Such inconsistencies in reported gene expression may reflect a highly dynamic pattern, as is likely to be the case for *rho*, which follows the pattern of EGF activity. Indeed, this is perfectly captured by our model. Alternatively, or concurrently, these inconsistencies could also reflect the difficulty to stage egg chambers with great precision following the canonical diagnostic features of Spradling [28]. Systematic co-expression experiments would be useful to devise a more precise timeline, in which the expression of each gene would be effectively linked to that of others. With our modeling approach, we can visualize the simulated expression pattern of any component of the system at any time point. Moreover, modeling offers a powerful way to evaluate the consistency of a proposed timeline with the underlying regulatory network. Our results thus provide a robust, consistent and, importantly, testable timeline of gene expression under such network architecture.

### Setting the posterior boundary to the appendage primordia

Another case of conflicting evidence bears on the influence of BMP activity on the anterior region. As discussed before, published data demonstrate a requirement for BMP activity for a cell to achieve roof-specific *br* expression [23], [43]. However, similar experiments in other publications have shown no effect of BMP signaling on Br outside of the anterior-most columnar cell rows [9], [42]. These results led to the proposal of a mechanism for defining the posterior boundary of the Br domains operating through EGF signaling at the posterior pole [18]. Importantly, the recent discovery of the role of Mid in eggshell patterning confirmed this function of early EGF signaling [24]. This model contrasts with the model where anterior Dpp sets this border in the follicular epithelium [23], [32].

In this paper we reconsider the earlier evidence in favor of BMP signaling in the putative DA primordia, and postulate an early BMP signal that precedes the known Dpp-driven activity in anterior follicle cells. An early signal (prior to stage 7, when the FCs stop dividing [28]) would generate different outcomes of mutation experiments disabling BMP signal transduction, depending on the stage at which they were induced (Figure 8), thus reconciling apparently conflicting results. Furthermore, we simulated early, mild overexpression of this signal and showed that this has a similar effect on eggshell patterning, and *br* expression in particular, as was observed experimentally using a GR1 Gal4 driver for *dpp* misexpression [43].

A similar misexpression experiment has been done with the BMP inhibitor Dad, using the CY2 Gal4 driver [42]. In this experiment, no effect was observed on the posterior border of the Br domains. However, CY2 drives Gal4 starting at stage 8 [63], which is after our hypothesized early BMP signal and the establishment of the anterior competence region. GR1, by contrast, is active in the FCs throughout oogenesis [75]. Thus, these results would also be consistent with an early BMP signal.

Mid has been demonstrated to establish an anterior competence region by repressing dorsal fate in posterior FCs [24]. We propose therefore that this early BMP signal could function through Mid, and likewise influence its expression. This proposal provides a clear hypothesis that could be tested experimentally, and given the onset of *mid* expression would effectively discern early from late BMP activity.

### The nature of the juxtacrine signal

In spite of the large body of knowledge gathered in the recent years, some important processes lack mechanistic explanations. This is the case for the formation of the roof-floor frontier, for which we hypothesize a juxtacrine mechanism. This signal, emanating from the Br-positive roof cells, has an instructive role for floor cell fate. Our model, with its underlying comprehensive network and extensive tests, helps defining important properties of this juxtacrine effect, and although many pieces are still missing in this puzzle, the hypotheses are clear and amenable to experimental dissection.

The putative mechanism behind this juxtacrine function requires at least two parts: a ligand expressed in the roof and a receptor expressed in, at least, the floor. Moreover, the signal should be able to lastingly enhance EGF activity. In our model, this mechanism is assigned to Br as a roof cell marker and to the unknown factor X, which relays the signal to the EGF signaling pathway. The positive effect of X over the EGF pathway is of particular importance after Grk extinction. Before this stage, though absence of X leads to a reduction of the size of the Br domain laterally and posteriorly, Grk activation of the EGF pathway is sufficient for *rho* expression. In contrast, X is required to maintain high dpERK levels in the floor domain after Grk extinction. This reduction in the precursors of the roof and the elimination of the floor domain predict the formation of thinner and shorter DAs [32].

As mentioned before, support for a juxtacrine signal in this system has been proposed by Simakov and colleagues [19], through a similar, albeit distinct, mechanism. These authors endorse a role for the transmembrane receptor Notch (represented by G5), which is proposed to activate Rho (G2) and repress Br (G3) in neighboring cells. However, Notch activity is known to repress Br cell-autonomously [22], not in neighboring cells as modeled by Simakov and colleagues, and the simulation of Notch mutants with this model ([19], Figure 4Ba) fails to capture the effect of Notch clones ([22], Figure 3A and B).

Notch is expressed strongly in the “T-region” in early stage 10 [22], [35], [56], and disappears from the T shortly thereafter [39]. Importantly, it borders the Br domains directly. While Notch is a viable candidate for the receptor in the proposed juxtacrine signal, information is lacking on the ligand. To the best of our knowledge, no Notch ligand specifically expressed in the roof has been identified. Therefore, it makes sense to examine other molecules and pathways that may perform this juxtacrine function, in addition to or perhaps in cooperation with Notch.

Initially proposed by Ward et al. [22] and later confirmed by Laplante and Nilson [76] to have a role in the formation of the floor-roof border, the cell-adhesion molecule Echinoid (Ed) is a Notch-interacting molecule. At late oogenesis, Ed is seen from stage 10B in the T domain, and later in the whole epithelium, except for the Br-positive cells marking the presumptive roof; interestingly, although Ed is present in the floor cells, it is absent from the floor membranes in contact with roof cells [24], [76]. Moreover, in the developing eye, Ed has been shown to antagonize EGF signaling [77]. If this same interaction occurs in the follicular epithelium, Ed could be a good candidate to be part of the juxtacrine mechanism.

To translate the postulated effect of Ed in our model, we need a mechanism that would influence dpERK activity in a manner consistent with Ed pattern, i.e. a mechanism that would result in comparatively weaker dpERK response outside of the roof domains and the immediately neighboring cells. In our model, this effect can be achieved by the extension of X activation to the roof domains. This results in a strong decrease in the number of roof cells (Figures 6B and 7B), as increased dpERK activates Pnt, which represses br. While this seems incongruent with our hypothesis, we note that high EGF activity does occur in the roof cells at stage 10B [17], not followed by the expected Pnt activation, an observation current models can not explain. This suggests that expression of *pnt* might be impaired in this domain: indeed, it is known that *br* expression itself disappears from the roof domains at the end of stage 10, even though the protein remains [42]. Alternatively, it may be that Br actively represses *pnt*, similar to the repression of the operculum and floor markers, Fas3 and *rho* [22], [56]. If confirmed, this would reconcile our model both with the experimental observations regarding high EGF activity in the roof, and with the hypothesis of Ed as a mediator for our juxtacrine effect. Thus, while the involvement of Notch is likely [22], we consider Ed to be another strong candidate for this role.

Finally, we believe that the software prototype specifically developed for this work may be used to qualitatively model other epithelial systems. The prospect of extending the current discrete framework to account for a better representation of long-range intercellular communication, further broadens the scope of the application. Moreover, it is conceivable that it may be transformed to account for cell proliferation and morphogenetic movements as pioneered in other studies [78], [79].

## Methods

### Single-cell models

At the cellular level, the models are developed using the logical formalism implemented in GINsim, a software freely available at http://ginsim.org [80]. GINsim supports the definition of logical regulatory networks and the construction of their dynamics. The software also provides tools to analyze these models, including the possibility to computationally determine all the stable states of a model [81].

A logical model consists of a regulatory graph and a set of logical rules. The graph is defined by a set of nodes, representing the regulatory components of the system, and by arcs, representing interactions. Input components, which are not regulated, account for external stimuli. Each component is identified to a variable that takes a limited number of discrete values. These values account for functional levels of the component's activity. Logical rules specify each component's target level depending on the levels of its regulators.

A state of the system is a vector whose elements are the components' levels. A state is *stable* if for all the components the target level equals the current level. Otherwise, the state is unstable and one or several variables are called to update. These updates define transition(s) leading to the successor state(s). When several variables are called to update, the number and identity of the successor state(s) depend on the updating scheme: synchronous (all the variables are simultaneously updated, defining a unique successor state), asynchronous (variables update independently, thus defining one successor for each updated variable), or user-defined through the use of priorities [82]. The set of states and transitions describe the (discrete) temporal evolution of the system, which is conveniently represented as a State Transition Graph. In this graph, stable states are nodes with no successor and oscillatory attractors are terminal strongly connected components (sets with no outgoing transitions and where every node is reachable from every other node through (a series) of edges [80]). Reachability analysis consists of assessing the existence of trajectories e.g. from one (initial) state to a stable state. Note that in the (deterministic) synchronous update a unique attractor is reachable from a given state, whereas in the asynchronous update, alternative trajectories may lead to different attractors.

### Epithelial models

As an epithelium, the system on which dorsal patterning of the eggshell plays out consists of a modular assembly of cells. Except when mutant clones are used, all cells contain the same genetic elements and are indistinguishable at the onset of the patterning process. For each cell, its associated model defines the evolution of the gene expression, depending on the activity of components of the proper cell, of genes from neighboring cells, or of other external signals. These external signals are implemented through integration variables (see A, S and X in the single-cell model Figure 3), whose values depend on the states of neighboring cells. At the end of the process, cells may assume different fates (stable states), depending on which genes are expressed.

An epithelial model is thus defined as a cellular automaton with hexagonal cells. Each cell has six direct neighbors, except along the anterior and posterior borders: the grid forms a cylinder. Moreover, cells are assigned the same model, and logical rules are extended to depend on the levels of regulators either within the cell or within neighboring cells, at any distance determined by the modeler. Simulations are carried out synchronously, for all variables in all cells. Whenever needed, some variables may be assigned a priority (e.g. here dpERK is updated before all other variables).

Like others [19], and for the sake of simplicity, we assume that the cells are static (i.e. they neither proliferate, nor move).

This framework is implemented in the form of a Python prototype, which is available at http://ginsim.org/node/176/, together with the model files.

## Supporting Information

Figure S1 **Attractors of the mechanistic, single-cell model.** The model gives rise to 8 stable patterns of the internal components (cellular fates) named F1 to F8 and 3 cyclic attractors (CA), all described in the table. The 12 compatible combinations of Grk, Dpp and Mid values define as many regions of the epithelium: R1 to R12 (see Figure 5). In each region, there are 18 combinations of values for the remaining inputs Aos_ext, Br_adj and Rho_ext. The pie charts indicate proportions of these combinations that are compatible with the attractors. Strikingly, in some regions a unique stable pattern arises (e.g. R5), and in general, fixed values of Grk and Dpp restrict the number of compatible attractors. Cyclical attractors exist in 3 regions (R2–4, R6–7), for few values of the inputs.
(TIF)

Text S1 **Summary of published evidence in support of the nodes and their relationships at the core of our model (see** **Figure 3** **).**
(PDF)
