# Supplementary figures and images for: A Discrete Model of Drosophila Eggshell Patterning Reveals Cell-Autonomous and Juxtacrine Effects
Source: PLoS Comput Biol. 2014 Mar 27;10(3):e1003527. doi: 10.1371/journal.pcbi.1003527 (PMC3967936; doi:10.1371/journal.pcbi.1003527)

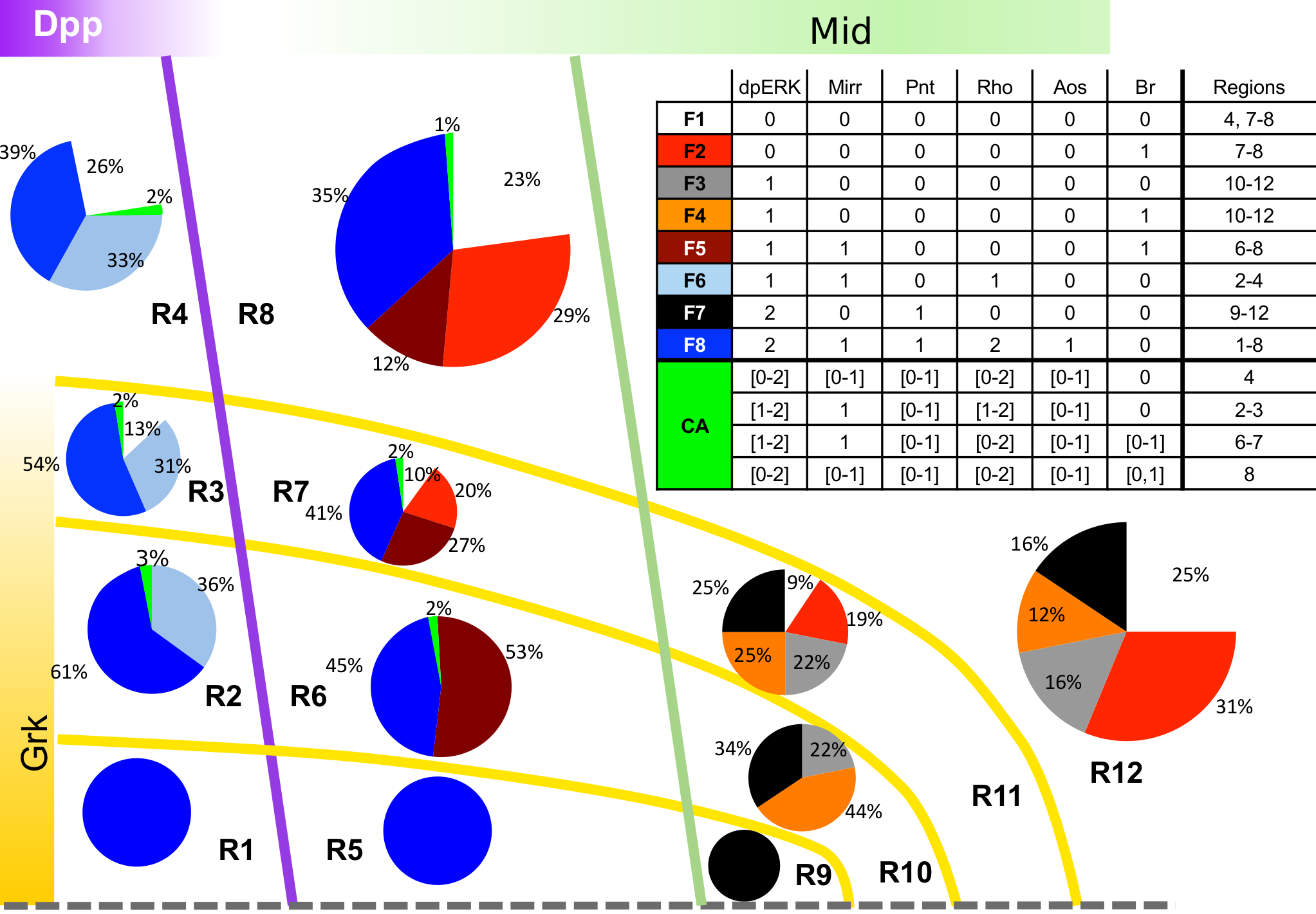

Supplement: Figure S1 — Attractors of the mechanistic, single-cell model. The model gives rise to 8 stable patterns of the internal components (cellular fates) named F1 to F8 and 3 cyclic attractors (CA), all described in the table. The 12 compatible combinations of Grk, Dpp and Mid values define as many regions of the epithelium: R1 to R12 (see Figure 5). In each region, there are 18 combinations of values for the remaining inputs Aos_ext, Br_adj and Rho_ext. The pie charts indicate proportions of these combinations that are compatible with the attractors. Strikingly, in some regions a unique stable pattern arises (e.g. R5), and in general, fixed values of Grk and Dpp restrict the number of compatible attractors. Cyclical attractors exist in 3 regions (R2–4, R6–7), for few values of the inputs. (TIF) [file pcbi.1003527.s001.tif]
