# Supplementary material for: A Discrete Model of Drosophila Eggshell Patterning Reveals Cell-Autonomous and Juxtacrine Effects
Source: PLoS Comput Biol. 2014 Mar 27;10(3):e1003527. doi: 10.1371/journal.pcbi.1003527 (PMC3967936; doi:10.1371/journal.pcbi.1003527)
Supplement: Text S1 — Summary of published evidence in support of the nodes and their relationships at the core of our model (see Figure 3 ). (PDF) [file pcbi.1003527.s002.pdf]

Documentation of the mechanistic, single-cell model

This supplementary material provides a summary of published evidence in support of the nodes and their relationships at the core of our model (Figure 3).

Input nodes

These nodes embody external influences and have no associated logical function.

Dpp
Decapentaplegic is a BMP ligand (a TGF-β superfamily member), secreted from the stretched FCs that overlay the nurse cells, and from stage 10 onwards in the centripetally migrating follicle cells [1]. It signals to the follicular epithelium through receptors Wit [30] and Tkv.
At stage 10, Dpp/BMP signaling activity can be detected in a narrow band along the anterior border of the follicular epithelium, slightly larger at the dorsal side [2,3].

Grk
Gurken is a TGF-α like protein secreted by the oocyte. It accumulates around the oocyte nucleus in the dorsal-anterior corner from stage 8 [4,5] and signals to the overlying follicular epithelium until the formation of the vitelline membrane at stage 10B [6].
Br_adj
This node denotes Br presence in neighboring cells (only those directly adjacent).
Aos_ext
This node denotes Aos presence in neighboring cells.
Rho_ext
This node denotes Rho presence in neighboring cells.
earlyBMP
dpp expression is first detectable at the end of stage 8 in approximately 20 to 30 somatic follicle cells at the anterior tip of the egg chamber [1]. However, there is evidence that the BMP pathway is active in early (<stage 6) egg chambers [7].
earlyEGF
Gurken is a TGF-α like protein secreted by the oocyte. In early stages, it is located in the posterior pole [4,5], where it activates EGF signaling in the overlying follicle cells.

Core nodes

The behavior of these "internal nodes" depends on other nodes (described in the bullet lists) following rules given in Figure 3.

Aos
argos expression begins around stage 10A in the dorsal centripetal follicle cells, before extending to the dorsal midline. The patch splits around stage 11-12 into a pattern reminiscent of the late pattern of *rho* [6,8–10]. It is targeted by

- Pnt, which induces *aos* in the neuroectoderm [11].
  Overexpression of *pntP1* may reduce expression of EGF signaling targets *rhomboid* and *69D* (unpublished data mentioned by [12]).
  Pnt may induce *aos* in the FCE (unpublished data mentioned by [6]).
- dpERK: in addition to the *argos* expression pattern, we know that in intervein cells in the wing imaginal disc, *aos* transcription is repressed by Cic in a cell autonomous way; Cic itself is targeted for degradation through the EGF signaling pathway [3,13–15].
- Mid: when EGFR is ectopically activated in the epithelium, *aos* transcription is restricted to the anterior side [8], which suggests repression by Mid.

Br

Broad-Complex (Br-C), is a transcription factor. It is a key marker of the roof region, expressed in two large patches of cells on each side of the dorsal midline [2,7,16–21]. *broad* mRNA is initially present at low levels everywhere, then repressed in an antero-dorsal T-shaped domain, before increasing in the roof domains and finally decreasing everywhere else.

br expression is controlled by two promoters [22]; only the BrL promoter is considered here.

- Mirr is required both to upregulate Br-C in the normal appendage primordium, and to downregulate it in the entire dorsal epithelium [20]. Fuchs et al. showed that Mirr activates the BrL promoter (late, Roof-specific Br pattern) and represses the BrE promoter (Early, ubiquitous Br pattern) [22].
- Pnt represses BrL (promoter controlling *broad* late expression) [22].

dpERK

Activation of the EGF pathway may be visualized directly through MAPK or ERK phosphorylation (indicated with dpERK), starting from a midline-centered pattern, which expands to encompass a larger part of the dorsal-anterior domain, before regressing to a spectacle-like pattern and finally to the L-shape pattern characteristic of the floor domain [6,9,10,21]. It is targeted by

- Gkr activates EGFR [4,8].
- Argos inhibits EGFR signaling by sequestering Spitz [23]. Secreted Spitz (after being cleaved by Rhomboid) activates EGFR [24–26].
- X, corresponding to a hypothetical enhancement of EGFR activity via a juxtacrine signal from the roof.

Mid

Midline expression marks the posterior domain of the follicular epithelium [27].

- earlyEGF: early, posterior Grk signalling activates Mid expression [27].
- earlyBMP: there is indirect evidence that BMP signaling affects the posterior boundary of the appendage primordia [8,18,28]. Considering the contrasting results of BMP pathway disruption on *br* expression [3,28–30], we postulate that this effect has to occur early, and propose it functions through repression of *mid*.

Mirr

The transcription factor Mirror is activated in a large dorsal-anterior patch of the epithelium by low levels of EGFR activity [28,31,32].

- dpERK; *mirr* is induced in a large dorsal-anterior patch of the epithelium by low levels of EGF activity through Cic [14,28,31,32].
- Mid represses *mirr* and its target *br* [27].

Pnt

The gene encoding the transcription factor Pointed is expressed along the dorsal midline at stage 10A, and in the roof region at stages 10B-11 [3,12].

- dpERK: *pnt* is induced by high levels of EGF signaling activity [2,11,12].

Rho

rho expression pattern seems to correlate with EGF signaling activity as it evolves from the whole dorsal-anterior region in early stages to the characteristic one-cell wide floor region, adjacent to the roof, in late stages [4,8,33,34]. Rhomboid, a protease, cleaves Spitz, which is then secreted [25,26].

- Mirr: ectopic expression of *mirr* induces *rho* ectopic expression [31,35].
  There is evidence that some anterior clue is required to induce *rhomboid* [8,18,29], and *mirror* could relay that signal to Rho.
- Br, which is required cell-autonomously to repress *rho* expression. Broad binding sites are predicted in the promoter region of *rho* [36].
- dpERK: *rho* transcription is repressed by the transcription factor CF2 [34]; CF2 is downregulated by the EGFR pathway [37].
- Dpp signaling represses *br* in the anterior follicle cells, via pMAD; strong *dpp* overexpression represses Br entirely [2,3].

Integration nodes

A

Combines the values of Aos and Aos ext.

- Aos and Aos_ext: Argos is secreted [38]. The *argos* gene encodes a diffusible factor that regulates cell fate decisions in the Drosophila eye. Rule for the epithelial model:
  level 1: at least 5 cells among the proper cell and 126 neighbors (direct and or at a distance up to 6 cells) express Aos.

S

Combines the values of Rho and Rho_ext, to account for Spitz signaling.

- Rho and Rho_ext: Rho activates Spitz, which is then secreted [25,26]. Rules for the epithelial model:
  level 2: autocrine (Rho at level 2) or at least 6 cells over six (direct) neighbors
  level 1: autocrine (Rho at level 1) or between 2 and 5 cells among the six (direct) neighbors.

X

Hypothetical mechanism. EGFR activity seems to be enhanced in cells in contact with the roof region ("spectacle-shaped" pattern in [9,10,21]). Moreover, Rho is expressed along the high-Notch side of a high/low Notch border; ectopic *rho* expression may be induced by ectopic high/low Notch barriers generated by Notch-clones [36].

- Br_adj and Br. Br is taken as a marker of the roof. As such, it activates X in the neighboring cells, while inhibiting it in the roof itself.

References

1. Twombly V, Blackman RK, Jin H, Graff JM, Padgett RW, et al. (1996) The TGF-beta signaling pathway is essential for Drosophila oogenesis. Development 122: 1555–1565.
2. Deng WM, Bownes M (1997) Two signalling pathways specify localised expression of the Broad-Complex in Drosophila eggshell patterning and morphogenesis. Development 124: 4639–4647.
3. Yakoby N, Lembong J, Schüpbach T, Shvartsman SY (2008) Drosophila eggshell is patterned by sequential action of feedforward and feedback loops. Development 135: 343–351. doi:10.1242/dev.008920.
4. Neuman-Silberberg FS, Schupbach T (1994) Dorsoventral axis formation in Drosophila depends on the correct dosage of the gene gurken. Development 120: 2457–2463.
5. Neuman-Silberberg FS, Schüpbach T (1996) The Drosophila TGF-alpha-like protein Gurken: expression and cellular localization during Drosophila oogenesis. Mech Dev 59: 105–113.
6. Wasserman JD, Freeman M (1998) An autoregulatory cascade of EGF receptor signaling patterns the Drosophila egg. Cell 95: 355–364.
7. Chen Y, Schüpbach T (2006) The role of brinker in eggshell patterning. Mech Dev 123: 395–406. doi:10.1016/j.mod.2006.03.007.
8. Queenan AM, Ghabrial A, Schüpbach T (1997) Ectopic activation of torpedo/Egfr, a Drosophila receptor tyrosine kinase, dorsalizes both the eggshell and the embryo. Development 124: 3871–3880.
9. Peri F, Bökel C, Roth S (1999) Local Gurken signaling and dynamic MAPK activation during Drosophila oogenesis. Mech Dev 81: 75–88.
10. Nakamura Y, Matsuno K (2003) Species-specific activation of EGF receptor signaling underlies evolutionary diversity in the dorsal appendage number of the genus Drosophila eggshells. Mech Dev 120: 897–907. doi:10.1016/S0925-4773(03)00164-3.
11. Chang D, Xu N, Luo K (2003) Degradation of cyclin B is required for the onset of anaphase in Mammalian cells. J Biol Chem 278: 37865–37873. doi:10.1074/jbc.M306376200.

12. Morimoto AM, Jordan KC, Tietze K, Britton JS, O'Neill EM, et al. (1996) Pointed, an ETS domain transcription factor, negatively regulates the EGF receptor pathway in Drosophila oogenesis. Development 122: 3745–3754.

13. Roch F, Jiménez G, Casanova J (2002) EGFR signalling inhibits Capicua-dependent repression during specification of ıt Drosophila wing veins. Development 129: 993–1002.

14. Atkey MR, Lachance J-FB, Walczak M, Rebello T, Nilson LA (2006) Capicua regulates follicle cell fate in the Drosophila ovary through repression of mirror. Development 133: 2115–2123. doi:10.1242/dev.02369.

15. Astigarraga S, Grossman R, Díaz-Delfín J, Caelles C, Paroush Z, et al. (2007) A MAPK docking site is critical for downregulation of Capicua by Torso and EGFR RTK signaling. EMBO J 26: 668–677. doi:10.1038/sj.emboj.7601532.

16. Tzolovsky G, Deng WM, Schlitt T, Bownes M (1999) The function of the broad-complex during Drosophila melanogaster oogenesis. Genetics 153: 1371–1383.

17. Dequier E, Souid S, Pál M, Maróy P, Lepesant JA, et al. (2001) Top-DER- and Dpp-dependent requirements for the Drosophila fos/kayak gene in follicular epithelium morphogenesis. Mech Dev 106: 47–60.

18. Ward EJ, Berg CA (2005) Juxtaposition between two cell types is necessary for dorsal appendage tube formation. Mech Dev 122: 241–255. doi:10.1016/j.mod.2004.10.006.

19. Yakoby N, Bristow CA, Gong D, Schafer X, Lembong J, et al. (2008) A combinatorial code for pattern formation in Drosophila oogenesis. Dev Cell 15: 725–737. doi:10.1016/j.devcel.2008.09.008.

20. Boisclair Lachance J-F, Fregoso Lomas M, Eleiche A, Bouchard Kerr P, Nilson LA (2009) Graded Egfr activity patterns the Drosophila eggshell independently of autocrine feedback. Development 136: 2893–2902. doi:10.1242/dev.036103.

21. Zartman JJ, Kanodia JS, Cheung LS, Shvartsman SY (2009) Feedback control of the EGFR signaling gradient: superposition of domain-splitting events in Drosophila oogenesis. Development 136: 2903–2911. doi:10.1242/dev.039545.

22. Fuchs A, Cheung LS, Charbonnier E, Shvartsman SY, Pyrowolakis G (2012) Transcriptional interpretation of the EGF receptor signaling gradient. Proc Natl Acad Sci USA 109: 1572–1577. doi:10.1073/pnas.1115190109.

23. Klein DE, Nappi VM, Reeves GT, Shvartsman SY, Lemmon MA (2004) Argos inhibits epidermal growth factor receptor signalling by ligand sequestration. Nature 430: 1040–1044. doi:10.1038/nature02840.

24. Rutledge BJ, Zhang K, Bier E, Jan YN, Perrimon N (1992) The Drosophila spitz gene encodes a putative EGF-like growth factor involved in dorsal-ventral axis formation and neurogenesis. Genes Dev 6: 1503–1517.

25. Lee LA, Elfring LK, Bosco G, Orr-Weaver TL (2001) A genetic screen for suppressors and enhancers of the Drosophila PAN GU cell cycle kinase identifies cyclin B as a target. Genetics 158: 1545–1556.

26. Urban S, Lee JR, Freeman M (2001) Drosophila rhomboid-1 defines a family of putative intramembrane serine proteases. Cell 107: 173–182.

27. Fregoso Lomas M, Hails F, Lachance J-FB, Nilson LA (2013) Response to the dorsal anterior gradient of EGFR signaling in Drosophila oogenesis is prepatterned by earlier posterior EGFR activation. Cell Reports 4: 791–802. doi:10.1016/j.celrep.2013.07.038.

28. Shravage BV, Altmann G, Technau M, Roth S (2007) The role of Dpp and its inhibitors during eggshell patterning in Drosophila. Development 134: 2261–2271. doi:10.1242/dev.02856.

29. Peri F, Roth S (2000) Combined activities of Gurken and decapentaplegic specify dorsal chorion structures of the Drosophila egg. Development 127: 841–850.

30. Marmion RA, Jevtic M, Springhorn A, Pyrowolakis G, Yakoby N (2013) The Drosophila BMPRII, wishful thinking, is required for eggshell patterning. Dev Biol 375: 45–53. doi:10.1016/j.ydbio.2012.12.011.

31. Jordan KC, Clegg NJ, Blasi JA, Morimoto AM, Sen J, et al. (2000) The homeobox gene mirror links EGF signalling to embryonic dorso-ventral axis formation through notch activation. Nat Genet 24: 429–433. doi:10.1038/74294.

32. Zhao D, Woolner S, Bownes M (2000) The Mirror transcription factor links signalling pathways in Drosophila oogenesis. Dev Genes Evol 210: 449–457. doi:10.1007/s004270050332.

33. Ruohola-Baker H, Grell E, Chou TB, Baker D, Jan LY, et al. (1993) Spatially localized rhomboid is required for establishment of the dorsal-ventral axis in Drosophila oogenesis. Cell 73: 953–965.

34. Hsu T, Bagni C, Sutherland JD, Kafatos FC (1996) The transcriptional factor CF2 is a mediator of EGF-R-activated dorsoventral patterning in Drosophila oogenesis. Genes Dev 10: 1411–1421.

doi:10.1101/gad.10.11.1411.

35. Jordan KC, Hatfield SD, Tworoger M, Ward EJ, Fischer KA, et al. (2005) Genome wide analysis of transcript levels after perturbation of the EGFR pathway in the Drosophila ovary. Dev Dyn 232: 709–724. doi:10.1002/dvdy.20318.

36. Ward EJ, Zhou X, Riddiford LM, Berg CA, Ruohola-Baker H (2006) Border of Notch activity establishes a boundary between the two dorsal appendage tube cell types. Dev Biol 297: 461–470. doi:10.1016/j.ydbio.2006.05.021.

37. Mantrova EY, Hsu T (1998) Down-regulation of transcription factor CF2 by Drosophila Ras/MAP kinase signaling in oogenesis: cytoplasmic retention and degradation. Genes Dev 12: 1166–1175.

38. Freeman M, Klämbt C, Goodman CS, Rubin GM (1992) The argos gene encodes a diffusible factor that regulates cell fate decisions in the Drosophila eye. Cell 69: 963–975.
